# Precision Medicine in Pediatric Cancer: Current Applications and Future Prospects

**DOI:** 10.3390/ht7040039

**Published:** 2018-12-13

**Authors:** Atif A. Ahmed, Divya S. Vundamati, Midhat S. Farooqi, Erin Guest

**Affiliations:** 1 Departments of Pathology and Pediatric Hematology-Oncology, University of Missouri School of Medicine, Kansas City, MO 64108, USA; dsvr5b@mail.umkc.edu (D.S.V.); msfarooqi@cmh.edu (M.S.F.); eguest@cmh.edu (E.G.); 2 Children’s Mercy Hospital, Kansas City, MO 64108, USA

**Keywords:** actionable mutations, next generation sequencing, pediatric cancer, precision medicine

## Abstract

Precision oncologic medicine is an emerging approach for cancer treatment that has recently taken giant steps in solid clinical practice. Recent advances in molecular diagnostics that can analyze the individual tumor’s variability in genes have provided greater understanding and additional strategies to treat cancers. Although tumors can be tested by several molecular methods, the use of next-generation sequencing (NGS) has greatly facilitated our understanding of pediatric cancer and identified additional therapeutic opportunities. Pediatric tumors have a different genetic make-up, with a fewer number of actionable targets than adult tumors. Nevertheless, precision oncology in the pediatric population has greatly improved the survival of patients with leukemia and solid tumors. This review discusses the current status of pediatric precision oncology and the different clinical scenarios in which it can be effectively applied.

## 1. Introduction

The management of patients with cancer can sometimes be challenging. These challenges are frequently attributed to tumor recurrence/metastasis during or following treatment or initial therapy resistance. In such situations, the use of novel targeted therapy has greatly revolutionized cancer treatment and is largely based on blocking actionable gene mutations or over-expressed signaling transduction pathways. Recent advances in molecular diagnostics have fueled the personalized approach to such cancer treatment. The fact that cancer is a genomic disease is the basis or foundation of precision oncology and targeted therapy [1,2]. Although early experiments on precision treatment started more than a century ago, only in the last decade has this term gained practical momentum in cancer treatment [3]. Precision medicine and its applications have started to fulfill their promise and their use have become more common in the last five years and in more than one cancer type [4].

Precision cancer treatment is largely based on matching the patient’s tumor mutations with the appropriate targeted therapy. A variety of genetic or molecular assays are currently available for testing on tumors including cytogenetic fluorescent in situ hybridization (FISH) tests, polymerase chain reaction (PCR) single gene testing, and single nucleotide polymorphism (SNP)-based microarrays [5]. In recent years, the practical use of such molecular tests in the diagnosis, risk assessment, response measurement and treatment of cancer patients has greatly expanded. However, the greatest benefit has come from next-generation sequencing (NGS) methods which include whole-genome sequencing (WGS), whole-exome sequencing (WES) and RNA sequencing (RNAseq), that offer many advantages over the single-drug, single-genetic test models [6]. NGS is a fast technology that allows for massively parallel sequencing of genomic fragments generating thousands to millions of short “reads” in a single run. It can detect point mutations including single nucleotide polymorphisms as well as small (generally, less than 20–30 base pairs) insertions/deletions (indels) in large numbers of genes at once. Coupled with powerful computational resources and tools to store, process and analyze the data, NGS can also identify unanticipated targetable mutations, copy number alterations, differentially expressed genes, and gene fusions [7]. The progressive decrease in the cost of sequencing (from more than $300,000 for an entire genome in 2008 to currently <$1000) and the increase in the number of genes tested in a typical sequencing panel (from 50 to over 400 genes) has allowed for the high yield extraction of data and presented enormous opportunities to study rare and difficult to treat neoplasms [8]. Other advantages of NGS are that it can be performed from a low amount of input DNA, which is beneficial when small needle biopsies are obtained, and that it can be performed on DNA extracted from formalin-fixed, paraffin-embedded tissue.

## 2. Pediatric Cancer Genome

Most cancers harbor somatic gene mutations, variants, or fusions that can be detected by NGS in clinically approved platforms. In a recent genetic analysis of 439 adult patients with cancer, 90% had at least one actionable or targetable mutation in their tumor [9]. In the pediatric population, precision oncology is an area of highly active research, with efforts focused on developing targeted therapies for patients who are not cured with standard treatment. Currently, however, the applications of molecular-based therapeutics in children are rather limited compared to the field of precision oncology for adult-onset cancers. Many of the advances in pediatric precision oncology thus far have been in leukemia, as opposed to solid tumors [10]. Pediatric malignancies are much rarer and are more often induced by inherited or sporadic errors in development rather than by environmental exposure. Thus the genomic landscape of alterations in pediatric cancer shows significant differences from adult cancers in terms of mutation frequency and type of altered genes [11]. With few exceptions, pediatric cancers exhibit a lower mutational burden with far fewer single nucleotide variants (SNVs) and small indels. On the other hand, childhood malignancies have a relatively high prevalence of specific structural variations (e.g., gene fusions and chromosomal rearrangements) and exhibit high specificity of associations with histologic tumor subtypes (see Table 1) [12,13]. Most of the genetic alterations in pediatric leukemias and solid tumors involve well-known genes and oncogenic pathways such as receptor tyrosine kinases, phosphoinositide 3-kinase-AKT and related pathways, *TP53*, *CDK12*, *NOTCH1*, *ARID1* and include amplification of other genes such as *MYCN*, *MCL1*, *MDM2* and others [14,15].

## 3. Precision Applications in Childhood Cancer

Several methods can be adopted for molecular precision analysis. Whole-exome, whole-genome, and RNA sequencing can be performed separately or complemented with methylation and expression microarray analysis. DNA methylation-based molecular diagnostics are currently being incorporated in the classification of pediatric central nervous system tumors [16]. An increasing number of gene-based targeted sequencing panels also are commercially available and are frequently used in clinical practice. Targeted sequencing improves the ability to detect low level clonal variants within a tumor, compared to WGS or WES studies [17].

**Figure 1:**
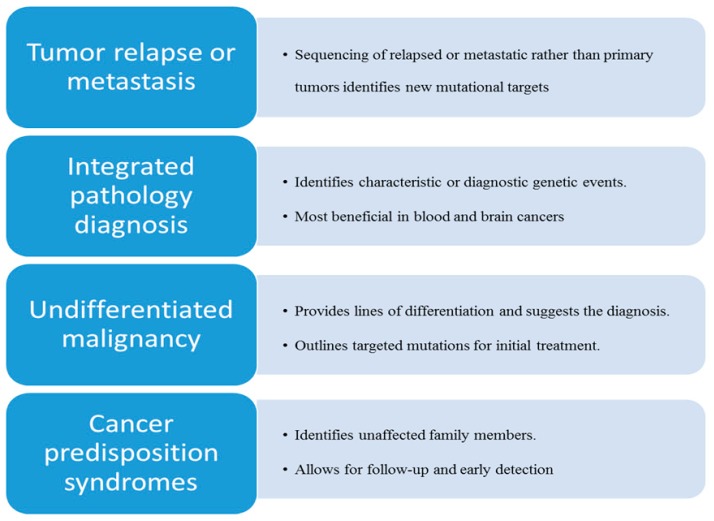
Most efficient use and benefits of next generation sequencing in pediatric cancer.

Although comprehensive molecular testing has been advocated for all pediatric tumors [18], it is also important to identify patient populations that may receive the maximal benefit from molecularly targeted therapy and accelerated development of novel drugs for early phase clinical trials. The most efficient clinical and practical use of tumor sequencing can be further highlighted in these specific scenarios (Figure 1):

### 3.1. Tumor Recurrence or Metastasis during Therapy

Pediatric cancers may occasionally recur or metastasize during or after completion of standard therapy. Relapsed tumors harbor more genetic alterations than primary tumors [11]. Tumor recurrence and metastasis have a significant adverse effect on the prognosis. When clinicians have exhausted all available standard treatment options, molecular precision methods can provide additional strategies. In some series, WES has provided clinically informative results in the majority (>90%) of adult patients with metastatic cancer [19,20]. In pediatric cancer, the number of actionable mutations has been reported to be much lower than that in adults, reflecting the different nature of these tumors. In a cohort of 91 young patients with relapsed or refractory cancer, only 42 patients (46%) had actionable findings that changed their cancer management [21]. Of 48 pediatric patients with recurrent or refractory cancer, clinically actionable findings were identified in 69% of patients, with the most efficient method being WES, followed by SNP array and RNAseq [22]. Despite the lower prevalence of actionable mutations, targeted therapy gives pediatric oncologists further hope in the treatment of refractory or relapsed pediatric cancers. In a case-controlled study of nine pediatric patients with refractory sarcoma, targeted therapy resulted in improvement of the overall survival to 8.83 months versus 4.93 months, and improvement of the progression-free survival to 6.17 months versus 1.6 months in the control group [23]. However, because of a possible increase in the number of mutations and changes in the mutational spectrum, the importance of testing relapsed or metastatic rather primary tumors cannot be over-emphasized [24].

Multidisciplinary management of cancers in an institutional molecular tumor board has led to better implementation or translation of NGS results into clinical actions for patients with refractory and poor prognosis cancers [25]. The major barriers to implementation of genomically guided therapy are the clinical status of the patient and access to off-label medications [25,26]. Reimbursement by third party payers either government-sponsored or from the private sector is also a major challenge to accessibility in many countries around the world [27].

### 3.2. Integrated Morphologic Molecular Diagnosis

The World Health Organization (WHO) diagnostic criteria for pediatric tumors, including most hematologic and brain tumors, are specified with integrated input from morphologic and molecular findings, reflecting the importance of molecular testing in clinical practice. Currently, many diagnostic genetic aberrations are tested by more routine molecular tests such as FISH and PCR. However, genomic sequencing is a sensitive technology for detecting diagnostic molecular aberrancies, can shed light on tumor heterogeneity and highlight differences (or similarities) between various pediatric and adult neoplasia. NGS can offer more accurate and comprehensive information on characteristic translocation partners, and identify targetable mutations that can lead to better treatments and cures. It has the potential to change or refine the morphologic diagnosis, offer prognostic information and direct care towards novel therapeutic agents [28]. After genomic profiling of 31 pediatric patients with brain tumors, the pathologic diagnosis was amended for 6 patients (19%), pathogenic germline mutations were detected, and potentially targetable alterations were identified in 19 patients (61%) [29]. A similar positive impact has been noted in pediatric hematologic malignancies. In 101 pediatric patients with high risk blood disorders tested with WES or RNAseq, clinically impactful findings were identified in 66% and potentially actionable mutations were present in 38% of cases [30]. In another cohort of 56 patients with high-risk hematologic malignancies and blood disorders, NGS findings contributed to the refinement of diagnosis and prognosis for 34% of patients [31]. Thus, routine testing of primary tumors by NGS is highly recommended for pediatric hematologic disorders and brain tumors and may be offered to other tumors as well.

### 3.3. Targeted Therapy of Undifferentiated Malignancies

In a rising number of cases, the treatment challenge stems from difficulties in classifying certain cancers that manifest with unusual or rare, non-characteristic morphology. Most often such cancers are labeled as undifferentiated malignancy or undifferentiated sarcoma after extensive histopathologic and immunophenotypic analysis. Undifferentiated sarcoma is commonly diagnosed when a soft-tissue tumor has no identifiable line of differentiation. These tumors represent a heterogeneous group of mesenchymal tumors with variable morphology that include spindle cell, pleomorphic, round cell, and epithelioid tumor variants. Undifferentiated sarcomas in children have variable outcomes and it is not possible to tailor the treatment to a specific disease type. In such patients, the routine use of NGS could increase diagnostic accuracy by determining the cell type of origin, identify molecularly-targeted therapies to try, and provide support for the use of other interventions, such as surgical resection and/or radiation therapy. Furthermore, it may help to better understand the patient’s prognosis for cure [32]. In the case of undifferentiated malignancies, early use of NGS can replace or decrease the number of other ancillary tests that are often requested during the processing of these tumors.

### 3.4. Cancer Predisposition Syndromes (CPS)

Recent studies indicate that a considerable percentage of childhood cancers are associated with CPS, some of which are inherited. The incidence of childhood cancers with genetic predisposition is estimated to be at least 10%, and the number of newly identified CPS has been steadily growing due to increased awareness and more practical use of NGS-based methods in the classification of pediatric cancer [33]. WES of parent-child trios has become an increasingly popular method to identify causative genetic variants in the germline of families with clustering of malignancies and metachronous tumors, and is a very powerful tool for providing unique insights into inheritance patterns. Trio testing can identify inherited versus *de novo* mutations, parental mosaicism, type of aberration (e.g., SNV, CNV, indels), and the dysregulation of cancer pathways (e.g., TP53, FA/BRCA) [34,35]. Tumors that fall into the category of CPS are diverse and include hamartomatous lesions, benign tumors, sarcomas, leukemia-lymphoma, and brain tumors (see Table 2) [36,37]. Clinicians and oncologists need to identify symptoms and signs of CPS correctly and refer patients for testing. NGS can also identify germline mutations in pre-symptomatic family members and allow for early detection of cancer through appropriate screening.

## 4. Future Prospects

Early trials of precision oncology have improved our understanding of chemoresistance during the course of therapy, and have highlighted cancer heterogeneity and genomic complexity, including the role(s) of epigenetic modifiers in determining disease outcome. NGS has revealed many cancers carry mutations in genes encoding for transcriptional control. Epigenetic dysfunction and transcriptional dysregulation is frequent in many pediatric cancers, particularly leukemias [38,39]. Inhibition of transcriptional programs and epigenetic modification are potential areas of opportunity in precision medicine. Combination therapy with simultaneous disruption of two genes, termed synthetic lethality, is emerging as a new avenue of targeted precision. This is based on the hypothesis that poor prognosis cancers with loss-of-function mutations become “treatable” when two otherwise discrete and unrelated genes are targeted simultaneously. Epigenetic regulators and genes involved in DNA repair are particularly attractive targets for cancer therapy because of their altered gene expression patterns in cancer cells, compared with normal cells. Targeting of these regulatory genes can selectively kill cancer cells [40,41].

Another potential and novel area of precision medicine is the use of nanotechnology to overcome treatment resistance of cancer cells [42]. Delivery systems based on nanoparticles enhance antitumor drug uptake and selective intracellular accumulation in the cancer cell. Multifunctional nanoparticles can deliver drug combinations for synergistic therapy, and facilitate personalization of therapeutic regimens [43]. Superparamagnetic iron oxide nanoparticles (SPIONs) have magnetic properties, show excellent tumor-targeting efficiency, and are thus more effective in personalized cancer treatment [44]. Thus, the use of nanotechnology with combination therapy, whether traditional or targeted, is an ideal model for personalized medicine and may hopefully result in cancer cure and eradication [45].

The use of antibodies against tumor antigens and stimulation of the patient’s own immune system to attack cancer cells are new forms of immunotherapy that have recently been used in patient treatments. A number of immunotherapy agents are in use or under investigation in pediatric cancer. Gemtuzumab ozogamicin has become part of the standard of care for pediatric patients with AML. Dinutuximab is an anti-GD2 antibody that is used in the treatment of neuroblastoma. Other immunotherapies under investigation include monoclonal antibodies (e.g., ganitumab), antibody-drug conjugates (e.g., brentuximab vedotin), bispecific T-cell engagers (e.g., blinatumomab), immune modulators (e.g., nivolumab, pembrolizumab, ipilimumab), and anti-cancer vaccines. Cellular therapies using modified T-cells have also been successfully employed. Based on their extraordinary ability to distinguish foreign peptides from self-antigens through their receptors, T cells are engineered with chimeric antigen receptors (CAR) designed for sustained proliferation and specific targeting of tumor cells. CAR-T cell therapy has been remarkably successful in treating patients with advanced refractory B cell malignancies through chimeric receptors targeting CD19 [46]. The success of CAR-T cell therapy in leukemia has been extrapolated to solid tumors where partial success has been demonstrated in sporadic case reports. A few clinical trials have reported using GD2-specific CAR-T cells for neuroblastoma and human epidermal growth factor receptor 2 (HER2) in medulloblastomas with non-dramatic results. A major challenge in CAR design is ensuring specificity for targeting tumor cells, while sparing healthy tissue and minimizing toxicity. Other major barriers need to be overcome for more successful CAR-T therapy. Reducing physical barriers in the extracellular matrix and eliminating the effect of an immunosuppressive microenvironment are some of the measures that ensure successful delivery of engineered T-cells to the tumor cells [47]. Although the outlook for CAR-T therapy promises superior benefits in the treatment of solid tumors, further progress is needed to overcome these challenges.

## 5. Conclusions

Several advances have been made in the last decade in the field of pediatric precision oncology heralding a new chapter in the fight against pediatric cancer. Although few pediatric institutions have started the initial journey towards precision oncology [48], several precision trials are now widely available for children with cancer. Publishing and sharing of clinical and genomic data will improve our understanding of targeted therapies leading to better patient management and further discoveries. The practical application of precision oncology in pediatric cancers is expected to grow exponentially with time and more investment in cancer research.

## Figures and Tables

**Table 1:** Genetic background of selected common pediatric tumors showing common structural rearrangements and recurrently mutated genes.

Tumor	Structural Rearrangements	Significantly Mutated Genes
Ewing’s sarcoma	*EWS-ETS*	*STAG2*, *CDKN2A*, *TP53*
Ewing’s-like round cell sarcoma	*CIC-FOX4*; *CIC-DUX4*; *BCOR-CCNB3*; *EWS-POU5F1/PATZI*	*CIC*, *BCOR*
Alveolar rhabdomyosarcoma	*PAX3/PAX7-FOXO1*; *PAX3-NCOA1*	*BCOR*, *PIK3CA*, *GAB1*, *PTEN*, *ARID1A*, *ROBO1*, *AKAP9*, *NEB*, *C15orf2*, *PTPRO*, *COL5A2*, *PXDNL*, *NLRC5*, *TTN*
Fusion-negative rhabdomyosarcoma	None	*FGFR4*, *RAS*, *AKT*, *PIK3CA*, *MYOD1*, *DICER1*, *CTNNB1*, *FBXW7*, *BCOR*, *TP53*
Osteosarcoma	None	*TP53*, *MDM2*, *RB1*, *ATRX*, *DLG2*, *PTEN*
Neuroblastoma	None	*ALK*, *MYCN* amplification, *ATRX*, *TERT*, *PTPN11*, *ARID1A*, *ARID1B*, *NF1*, *RAS*, *BRAF*, *FGFR1*
Wilms tumor	None	*WT1*, *CTNNB1*, *WTX*, *DICER1*, *DIS3L2*, *SIX1/2*, *MLLT1*, *TP53*, *FBXW7*, *MYCN*, *CTR9*, *REST*
Malignant rhabdoid tumor	None	*SMARCB1*, *SMARCA4*
Translocation renal cell carcinoma	Xp11 (TFE3)	*TFE*, *SMARCC2, KDM5C, INO80D, CHD, MLL3*
Clear cell sarcoma of the kidney	*YWHAE-NUTM2*	*BCOR* duplication
Synovial sarcoma	*SS18-SSX1/2*	*NGDN*, *RASAL3*, *KLHL34*, *MUM1L1*, *EP300*
Dermatofibrosarcoma protuberans	*COL1A1-PDGFB*	*CARD10*, *PPP1R39*, *SAFB2*, *STARD9*

More complete information can be found in the Childhood Cancer Genomics PDQ [15].

**Table 2:** Mutated genes and dysregulated signaling pathways in selected common cancer predisposition syndromes (see reference [37]).

Cancer Predisposition Syndrome	Common Tumors	Mutated Genes	Dysregulated Pathways and Functions
Li Fraumeni syndrome	Leukemias, osteosarcoma, soft tissue sarcoma, adrenocortical, brain tumors	*TP53*, *CHEK2*	Cell cycle, apoptosis
Familial adenomatous polyposis	Colonic polyps, osteomas, desmoid, thyroid and adrenal tumors	*CTNNB1*, *MUTYH*	WNT/β-catenin
Fanconi anemia	Leukemias, hepatic tumors, upper airways carcinomas	*FANC* family of genes, *BRCA2*	DNA repair
Nevoid basal cell carcinoma	Basal cell carcinomas, jaw cysts	*PTCH*	Sonic hedgehog (SHH)
Neurofibromatosis	Peripheral nerve and nerve sheath tumors, brain gliomas, GIST tumors	*NF1*	GTPase, RAS/MAPK inactivation
Von Hippel–Lindau	Renal carcinoma, hemangioblastoma, pheochromocytoma, pancreatic tumors	*VHL*	HIF degradation, RNA polymerase II regulation
Dicer1 syndrome	Pleuropulmonary blastoma	*Dicer1*	miRNA synthesis
Xeroderma pigmentosum	Skin cancers	*XP* family of genes	DNA repair
Hereditary paraganglioma-pheochromocytoma	Paraganglioma, pheochromocytoma, renal cell carcinoma	*SDH*	Citric acid cycle, oxidative phosphorylation
Ataxia telangiectasia	Leukemia, lymphoma	*ATM*	DNA repair
Bloom syndrome	Leukemia, lymphoma	*BLM*	DNA repair
PTEN hamartoma	Breast, thyroid, GI polyps	*PTEN*	Tyrosine kinase de-phosphorylation
Hamartomatous polyposis (Peutz-Jeghers/juvenile polyposis)	Gastrointestinal polyps	*LKB1/STK11*; *SMAD4*; *BMPR1A*; *ENG*	DNA mismatch repair
Hereditary non-polyposis colorectal cancer	Colorectal cancer (very rare in children)	*MLH1*, *MSH2*, *MSH6*, *or PMS2*	DNA mismatch repair
